# A phase Ib study of the combination regorafenib with PF-03446962 in patients with refractory metastatic colorectal cancer (REGAL-1 trial)

**DOI:** 10.1007/s00280-019-03916-0

**Published:** 2019-08-23

**Authors:** Jeffrey Melson Clarke, Gerard C. Blobe, John H. Strickler, Hope Elizabeth Uronis, S. Yousuf Zafar, Michael Morse, Evan Dropkin, Leigh Howard, Margot O’Neill, Christel N. Rushing, Donna Niedzwiecki, Hollie Watson, Emily Bolch, Christy Arrowood, Yingmiao Liu, Andrew B. Nixon, Herbert I. Hurwitz

**Affiliations:** 1 Duke Cancer Institute, Duke University Medical Center, 20 Medicine Circle, Morris Building, Rm 25178, DUMC Box 3198, Durham, NC 27710 USA; 2 Genentech, Inc, San Francisco, CA 94080 USA

**Keywords:** Regorafenib, PF-03446962, Phase Ib, Colorectal cancer, ALK-1

## Abstract

**Purpose:**

This study aimed to evaluate the maximum tolerated dose (MTD) and recommended phase II dose (RPTD), as well as the safety and tolerability of PF-03446962, a monoclonal antibody targeting activin receptor like kinase 1 (ALK-1), in combination with regorafenib in patients with refractory metastatic colorectal cancer.

**Methods:**

The first stage of this study was a standard “3 + 3” open-label dose-escalation scheme. Cohorts of 3–6 subjects were started with 120 mg of regorafenib given PO daily for 3 weeks of a 4 week cycle, plus 4.5 mg/kg of PF-03446962 given IV every 2 weeks. Doses of both drugs were adjusted according to dose-limiting toxicities (DLT). Plasma was collected for multiplexed ELISA analysis of factors related to tumor growth and angiogenesis.

**Results:**

Seventeen subjects were enrolled, of whom 11 were deemed evaluable. Seven subjects were enrolled at dose level 1, and four were enrolled at level − 1. Overall, three DLTs were observed during the dose-escalation phase: two in level 1 and one in level − 1. A planned dose-expansion cohort was not started due to early termination of the clinical trial. Common adverse events were infusion-related reaction, fatigue, palmar-plantar erythrodysesthesia syndrome, abdominal pain, dehydration, nausea, back pain, anorexia, and diarrhea. One subject achieved stable disease for 5.5 months, but discontinued treatment due to adverse events.

**Conclusions:**

The regimen of regorafenib and PF-03446962 was associated with unacceptable toxicity and did not demonstrate notable clinical activity in patients with refractory metastatic colorectal cancer.

## Introduction

Vascular endothelial growth factor A (VEGF-A) and VEGF receptor-2 (VEGFR2) together are considered major drivers of angiogenesis. Current anti-angiogenic therapies involve monoclonal antibodies and ligand traps targeting either VEGF-A or VEGFR2 or small-molecule receptor tyrosine kinase inhibitors of VEGFRs. While these approaches have been successful at improving survival in a number of malignancies (i.e., colorectal, hepatocellular, renal cell, and non-small cell lung carcinomas), disease progression inevitably occurs. A number of angiogenic factors and specific molecular pathways have been implicated in controlling the mechanisms of therapeutic resistance with these anti-angiogenic agents [1]. Targeting these resistance mechanisms with novel anti-angiogenic agents may potentiate treatment response when used in combination or in sequence with VEGF/VEGFR-directed therapies.

Specifically, novel agents inhibiting TGF-β signaling may potentiate treatment response when used in combination with VEGF/VEGFR-directed therapies. In particular, the TGF-β axis may provide an escape pathway for tumor angiogenesis [2]. TGF-β1 and BMP-9 are ligands for type II TGF-β receptors and CD105 (endoglin), which form heterotetrameric complexes with type I receptors activating an intracellular signaling cascade via phosphorylation of Smad proteins 1/5/8. A predominance of Smad 1/5/8 activation results in up-regulation of multiple genes, stimulating endothelial cell proliferation, migration, survival, and tube formation and culminating in the activation phase of angiogenesis [3]. Activin receptor like kinase 1 (ALK-1) is a TGF-β transmembrane receptor with restricted expression to proliferating endothelial cells and is critical to TGF-β-mediated angiogenesis [2]. ALK-1 expression is detected on vascular endothelium and circulating endothelial cells in numerous malignancies including breast, prostate, renal cell, and colorectal carcinomas [4]. Importantly, VEGF-A and ALK-1 expression is known to be interrelated in regulating angiogenesis [2].

PF-03446962 is an IgG2 monoclonal antibody targeting human ALK-1, which in preclinical studies has demonstrated enhanced tumor growth inhibition in combination with VEGF axis blockade by either monoclonal antibody or TKI inhibitor in tumor xenografts that were resistant to anti-angiogenic therapy [4]. PF-03446962 has been evaluated in multiple early phase clinical trials in advanced solid tumors, urothelial cell carcinoma, hepatocellular carcinoma, and mesothelioma [5–9]. As a monotherapy, PF-03446962 was generally associated with limited clinical efficacy. However, we hypothesized that targeting of the VEGF axis in combination with a complimentary pathway of angiogenesis and potential mechanism of resistance would result in synergistic inhibition of tumor growth and improve clinical outcomes. To evaluate this hypothesis, we conducted a phase Ib clinical trial of PF-03446962 in combination with regorafenib in patients with advanced refractory colorectal cancer.

## Patients and methods

### Study design

This was a dose-escalation phase Ib and biomarker study to assess the combination of regorafenib (Bayer Health Care Pharmaceuticals, Inc) and PF-03446962 (Pfizer, Inc.) in patients with refractory metastatic colorectal cancer. A standard “3 + 3” dose escalation was used to establish the MTD/RPTD of regorafenib and PF-03446962. The MTD was defined around toxicities in the first 28 day cycle. There was a planned escalation cohort of 6–15 additional subjects planned to enroll at the RPTD to assess safety and tolerability; however, this cohort was not opened. The cycles were 28 days long, and treatment continued until disease progression, patient refusal, or unacceptable toxicity, whichever occurred first. Patients were first treated with 120 mg of regorafenib and 4.5 mg/kg of PF-03446962. Once two subjects experienced DLT, the dose level was lowered to 3 mg/kg of PF-03446962. Archived paraffin-embedded tissue and blood were collected for future analysis of factors related to tumor growth and angiogenesis that may predict response, resistance, or toxicity.

### Patients

Eligible patients were required to have histologically and/or cytologically confirmed and radiographically evaluable refractory metastatic colorectal adenocarcinoma for which regorafenib would be considered a therapeutic option. Additional inclusion criteria included: measureable disease per RECIST 1.1 criteria; age ≥ 18 years; ECOG 0 or 1; life expectancy of at least 3 months; adequate bone marrow function (as shown by ANC ≥ 1.5 × 10^9^, platelets ≥ 100 × 10^9^/L, Hgb ≥ 9 g/dL); adequate liver function (as shown by serum bilirubin ≤ 1.5 × ULN, PT/PTT/INR ≤ 1.5 × ULN; ALT and AST ≤ 2.5 × ULN); adequate renal function (CrCl ≥ 50 cc/min by Cockroft Gault or 24 h urine; presence of an archived tumor sample. Exclusion criteria included: prior failure to tolerate regorafenib at 120 mg/day; current or previous anticancer therapy within 4 weeks from D1 of study drug; major surgery or traumatic injury within 4 weeks from D1 of study drug; not recovering from the side effects of any major surgery; anticipated to have major surgery during the course of the study; hypersensitivity reactions to regorafenib and/or structural compound, biological agent, or formulation; grade 3–4 AE associated with prior anti-VEGF therapy; history of grade 3 or higher hypersensitivity attributed to humanized and/or chimeric monoclonal antibodies; patients receiving chronic, systemic treatment with corticosteroids; active brain or leptomeningeal metastases; severe COPD or other pulmonary disease with hypoxemia; poorly controlled atrial fibrillation; previous history of CVA, TIA, angina pectoris, acute MI, or history of recent re-perfusion procedures, pulmonary embolus, or untreated DVT within 6 months from start of study drug; known CAD or PVD or CVD; congestive heart failure (NYHA classification III–IV); proteinuria at screening (UA > 1+ and 24 h urine protein ≥ 1 g/24 h); severely impaired lung function; active or uncontrolled severe infection requiring treatment with IV antibiotics; liver disease; poorly controlled hypertension; impaired GI function that may significantly alter absorption of oral medications; significant vascular disease; history of hemoptysis; history of abdominal fistula or GI perforation within 6 months prior to D1 of study drug; active peptic ulcer disease, IBD, or other GI condition with increased risk of perforation or GI bleeding; use or need for full-dose anticoagulation; serious, non-healing wound, active ulcer, or untreated bone fracture; active bleeding diathesis; endobronchial lesions and/or lesions infiltrating major pulmonary vessels that increase the risk of pulmonary hemorrhage; known history of HIV, hepatitis B or C; female patients who are pregnant or breast feeding or adults of reproductive potential not willing to use effective birth control; concomitant use of CYP3A4 strong inducers or strong inhibitors; corrected QTc interval > 500 ms; history of Osler–Weber–Rendu syndrome or Hereditary Hemorrhagic Telangiectasia.

This was a single-center study approved by the Duke Institutional Review Board and following the guidelines of the Helsinki Declaration. All patients provided informed written consent prior to any study-related procedure and were treated at Duke Cancer Institute. Subject accrual took place from Sept 2014 to Nov 2015.

### Clinical and radiographic assessment

All subjects completed the following baseline assessments to determine eligibility prior to receiving study drug: extensive medical history, physical exam, vital signs, height and weight, ECOG performance status, baseline symptom assessment, CBC with differential, serum chemistry, pregnancy test (females of childbearing potential), PT/PTT/INR, phosphorus (if clinically indicated), TSH (if clinically indicated), amylase and lipase (if clinically indicated), urinalysis or UPCR, EKG, radiographic tumor assessment, tumor blood markers, and confirmation of archived paraffin tumor sample availability. Throughout the study treatment phase, subjects received all of the above listed assessments within 72 h prior of drug administration on D1 of the cycle, with the exception of height, EKG, radiographic tumor assessment, tumor blood markers, and confirmation of tumor availability. Subjects were also assessed for adverse events and concomitant medication administration throughout their participation in the study. On D15 of each cycle, subjects had the following assessments prior to infusion: vital signs, CBC with differential, serum chemistry, phosphorus (if clinically indicated), TSH (if clinically indicated), and amylase and lipase (if clinically indicated). Every 8 weeks, subjects were restaged and received and EKG, radiographic tumor assessment, and tumor blood markers.

Tumor response was assessed via computed tomography (CT) scan or magnetic resonance imaging (MRI) every 8 weeks using response evaluation criteria in solid tumor (RECIST) criteria (version 1.1). General symptom management and supportive care were provided as clinically indicated. Clinical activity was defined as complete response (CR), partial response (PR), or stable disease (SD).

### Safety

The NCI common toxicity criteria version 4.0 was used to grade adverse events. If study treatment-related and occurring during cycle 1, the following adverse events were considered to be DLTs: hematologic toxicity (defined as any grade 4 neutropenia, thrombocytopenia, or anemia, or grade ≥ 3 neutropenia or thrombocytopenia lasting over 7 days); any grade 3 thrombocytopenia associated with bleeding; neutropenic fever; nausea/vomiting/diarrhea of grade 3 or greater lasting 3 days or more despite adequate supportive care; grade ≥ 3 ALT or AST elevation > 7 days; other non-hematologic toxicity ≥ grade 3, excluding alopecia, anorexia, fatigue, hypertension, isolated (non-clinically significant) lab abnormalities, and/or rare, idiosyncratic reactions to any study drug; treatment delay of ≥ 14 days for cycle 2 due to unresolved treatment-related toxicity; less than 75% dose intensity of regorafenib. Patients were considered evaluable for toxicity if they received any study treatment and evaluable for DLT/MTD determinations if they completed cycle 1, or experienced DLT in cycle 1.

### Biomarkers

Double-spun, platelet poor plasma was collected at baseline, every restaging, and at the time the subject came off-treatment and stored at − 80 °C. Baseline and longitudinal samples were analyzed for factors related to tumor growth and angiogenesis, including 24 blood biomarkers (supplemental). All biomarkers were measured using the CiraScan multiplex platform (Aushon Biosystems, Inc., Billerica, MA, USA), except for BMP-9 and TGFβ-R3. TGFβ-R3 [10] and BMP-9 [11] were assessed as previously described.

### Statistics

The objectives of this study were to assess the MTD/RPTD of PF-03446962 in combination with regorafenib, describe the safety and tolerability profile, evaluate the clinical efficacy, and explore genetic changes potentially associated with treatment. The MTD was defined by the frequency of DLTs based on the standard “3 + 3” dose-escalation study design. Adverse events (AEs) summarized safety and tolerability using frequencies and percentages. Clinical efficacy was evaluated using tumor response, progression-free survival (PFS), and overall survival (OS). Tumor response was defined by RECIST criteria v 1.1 and summarized using frequencies and percentages. The endpoint for PFS was disease progression or death from any cause. The endpoint for OS was death from any cause. The medians and confidence intervals for OS and PFS were estimated using the Kaplan–Meier method.

Biomarker expression levels from samples collected at baseline (C1D1), during cycle 1 (C1D7, C1D15, or C1D21), and at the start of cycle 2 (C2D1) were described, the changes in expression evaluated, and the association between expression levels and response explored. Expression levels were described using medians and ranges. Changes in biomarker levels from earlier to later time points were assessed as the binary logarithm of the ratio of later to earlier and evaluated using Wilcoxon signed-rank tests; *p* values were not adjusted for multiple testing. The potential association between expression levels and response was explored graphically with no hypothesis testing performed. Biomarker analyses were performed in R v3.4 and all other statistical analyses completed using SAS v9.4.

## Results

Two dose levels were administered for the combination of regorafenib with PF-03446962, with seven evaluable subjects in dose level 1 (120 mg regorafenib + 4.5 mg/kg PF-03446962) and four evaluable subjects in dose level − 1 (120 mg regorafenib + 3 mg/kg PF-03446962). Enrollment was stopped early by the sponsor due to reprioritization of the PF-03446962 development program. Patient demographics are summarized in Table 1. A total of 17 subjects were enrolled, and of those, 11 were evaluable for toxicity. All subjects enrolled had metastatic colorectal cancer had received on average three prior lines of therapy (range 2–4).

**Table 1:** Demographics of patients enrolled in the phase II trial of regorafenib and PF-03446962

All percentages: column	Cohort				All		
	Level − 1		Level 1				
	*N*	%	*N*	%	*N*	%	
All	4	100.0	7	100.0	11		100.0
Age, mean (std)	50.4	17.0	62.8	13.4	58.3		15.3
Race							
Black or African–American	3	75.0	3	42.9	6		54.5
White	1	25.0	4	57.1	5		45.5
Ethnicity							
Not Hispanic or Latino	4	100.0	7	100.0	11		100.0
Gender							
Female	1	25.0	3	42.9	4		36.4
Male	3	75.0	4	57.1	7		63.6
Diagnosis							
Colon	4	100.0	6	85.7	10		90.9
Rectum	0	0.0	1	14.3	1		9.1

Two out of seven subjects enrolled at dose level 1 experienced DLT that was attributed as possibly related to study treatment. One of four subjects at dose level − 1 experienced a DLT; however, it was deemed unrelated to study treatment. One patient developed a grade 5 pelvic infection in dose level 1 that was deemed treatment-related (Tables 2 and 3). A grade 3 infusion reaction was noted in one patient on study, as well. Of note, the study was terminated prior to the MTD or recommended phase 2 dose being confirmed.

**Table 2:** Treatment-related AEs

All percents are for the cohort represented in that column	Cohort												All (*n* = 11)					
	Level − 1 (*n* = 4)						Level 1 (*n* = 7)											
	Grade						Grade						Grade					
	2		3		5		2		3		5		2		3		5	
	*N*	%	*N*	%	*N*	%	*N*	%	*N*	%	*N*	%	*N*	%	*N*	%	*N*	%
Adverse event																		
Abdominal pain	–	0	–	–	–	–	2	29	1	14	–	0	2	18	1	9	–	0
Anorexia	1	25	–	–	–	–	2	29	–	0	–	0	3	27	–	0	–	0
Back pain	–	0	–	–	–	–	1	14	–	0	–	0	1	9	–	0	–	0
Dehydration	–	0	–	–	–	–	3	43	–	0	–	0	3	27	–	0	–	0
Diarrhea	1	25	–	–	–	–	1	14	–	0	–	0	2	18	–	0	–	0
Fatigue	1	25	–	–	–	–	4	57	–	0	–	0	5	45	–	0	–	0
Infusion-related reaction	–	0	–	–	–	–	2	29	1	14	–	0	2	18	1	9	–	0
Nausea	1	25	–	–	–	–	1	14	–	0	–	0	2	18	–	0	–	0
Palmar-plantar erythrodysesthesia syndrome	1	25	–	–	–	–	3	43	–	0	–	0	4	36	–	0	–	0
Pelvic infection	–	0	–	–	–	–	–	0	–	0	1	14	–	0	–	0	1	9

**Table 3:** All reproted AEs

All percents are for the cohort represented in that column	Cohort												All (*n* = 11)					
	Level − 1 (*n *= 4)						Level 1 (*n* = 7)											
	Grade						Grade						Grade					
	2		3		5		2		3		5		2		3		5	
	*N*	%	*N*	%	*N*	%	*N*	%	*N*	%	*N*	%	*N*	%	*N*	%	*N*	%
Adverse event																		
Abdominal pain	1	25	–	–	–	0	2	29	1	14	–	0	3	27	1	9	–	0
Anorexia	3	75	–	–	–	0	2	29	–	0	–	0	5	45	–	0	–	0
Back pain	1	25	–	–	–	0	2	29	–	0	–	0	3	27	–	0	–	0
Death NOS	–	0	–	–	1	25	–	0	–	0	–	0	–	0	–	0	1	9
Dehydration	–	0	–	–	–	0	3	43	–	0	–	0	3	27	–	0	–	0
Diarrhea	2	50	–	–	–	0	1	14	–	0	–	0	3	27	–	0	–	0
Fatigue	1	25	–	–	–	0	4	57	–	0	–	0	5	45	–	0	–	0
Infusion-related reaction	–	0	–	–	–	0	2	29	1	14	–	0	2	18	1	9	–	0
Nausea	2	50	–	–	–	0	1	14	–	0	–	0	3	27	–	0	–	0
Palmar-plantar erythrodysesthesia syndrome	1	25	–	–	–	0	3	43	–	0	–	0	4	36	–	0	–	0
Pelvic infection	–	0	–	–	–	0	–	0	–	0	1	14	–	0	–	0	1	9

**Fig. 1:**
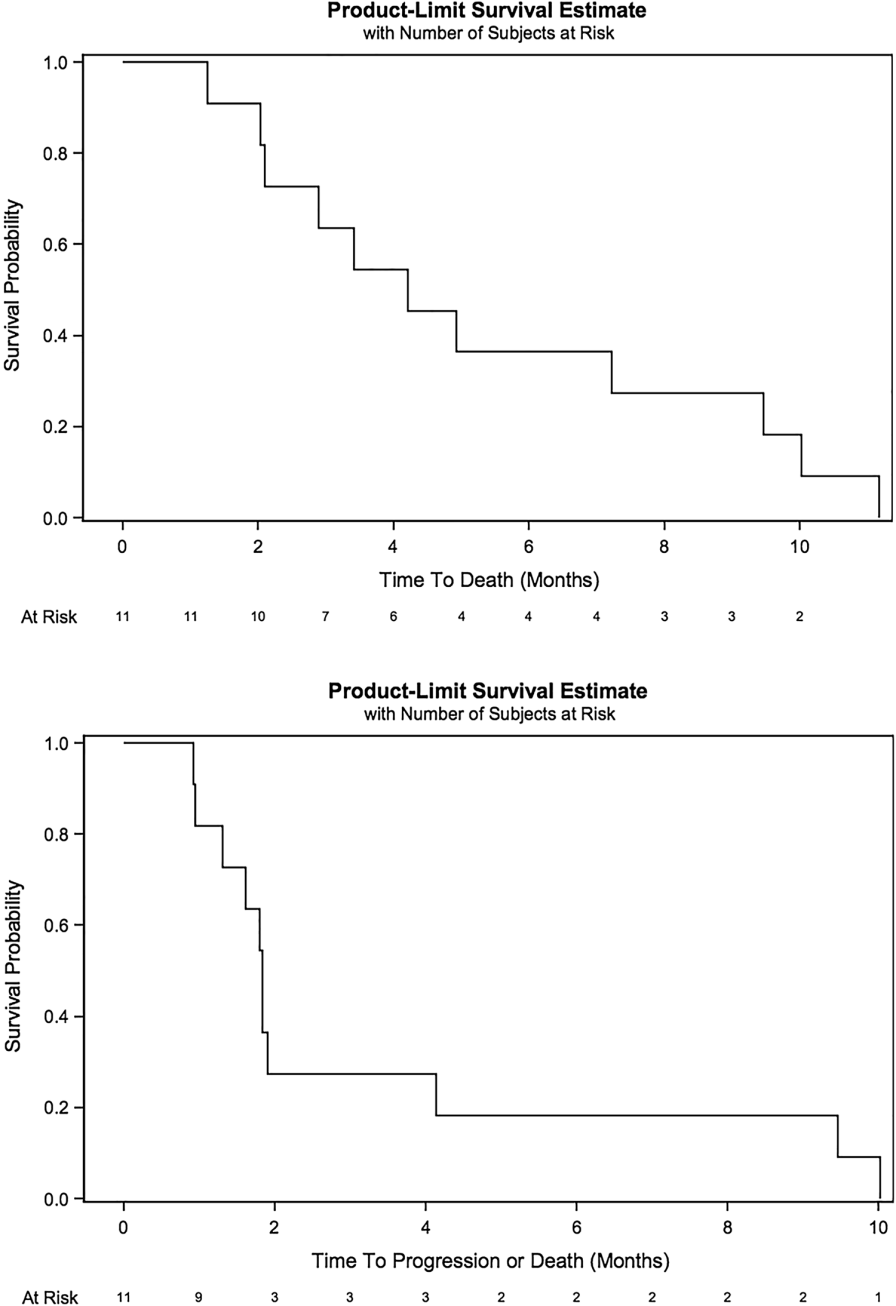
Progression-free survival (**a**) and overall survival (**b**) for patients treated with PF-03446962 and regorafenib

Of the 11 patients enrolled and treated in the clinical trial, 1 patient was deemed inevaluable for efficacy. Eight patients had developed progressive disease (72.7%) and two patients experienced stable disease (18.2%) (Table 4). Median progression-free survival was 1.84 months (95% CI 0.95–4.14) and median overall survival of 4.21 months (95% CI 2.04–9.46)  (Fig. 1a and b).

**Table 4:** Clinical outcomes including best overall response, progression free survival, and overall survival

	Cohort				All	
	Level − 1		Level 1			
	*N*	%	*N*	%	*N*	%
All	4	100.0	7	100.0	11	100.0
Best overall response						
Inevaluable	0	0.0	1	14.3	1	9.1
Progressive disease	4	100.0	4	57.1	8	72.7
Stable disease	0	0.0	2	28.6	2	18.2
Median OS (95% CI) in months	2.76	1.25–4.21	7.23	2.04–10.0	4.21	2.04–9.46
Median PFS (95% CI) in months	1.71	0.92–1.84	1.91	0.95–9.46	1.84	0.95–4.14

### Biomarkers

**Fig. 2:**
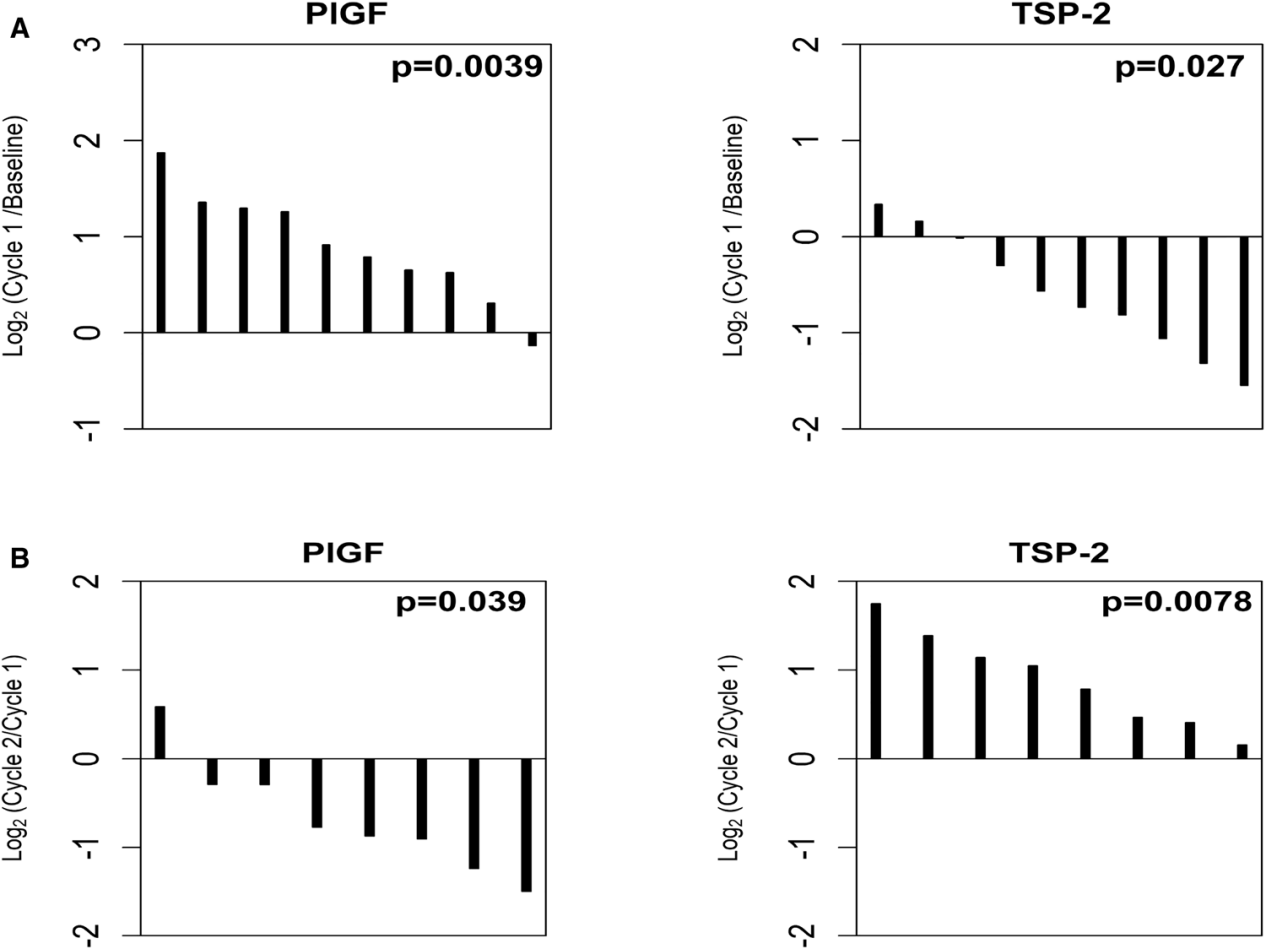
Biomarker change in response to Regorafenib and PF-03446962. **a** Early changes during cycle 1 of therapy and **b** late changes during cycle 2 of therapy

**Fig. 3:**
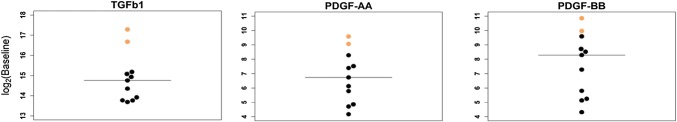
Baseline expression of TGF-β related markers in patients with stable disease (SD, gold dot) progressive disease (PD, black dot)

Plasma samples from all 11 patients were analyzed to assess the expression of 24 biomarkers related to angiogenesis and inflammation (Table 5). Markers were assessed at three time points, including baseline (prior to any treatment), during cycle 1 of therapy, and during cycle 2 of therapy. We noted that nine biomarkers (BMP-9, PlGF, TGF-β2, TGFβ-R3, TSP-2, VCAM-1, VEGF-A, VEGF-R2, and VEGF-R3) significantly changed from baseline during the first cycle of this regimen. As shown in Fig. 2a, PlGF increased (*p* = 0.0039), while TSP-2 decreased (*p* = 0.027) in the majority of patients. At later time points, we observed a reversal of these changes; levels of PlGF were decreased at cycle 2 compared to cycle 1 (*p* = 0.039) and levels of TSP-2 were increased at cycle 2 compared to cycle 1 (*p* = 0.0078) (Fig. 2b). Of the 11 patients evaluated, 2 patients exhibited SD, while 9 patients exhibited PD or were inevaluable. Dichotomizing the patients into these two groups (SD vs PD/inevaluable), we explored the potential relationship between biomarker expression and response. Due to the very small number of patients assessed, these results are considered hypothesis-generating only and highly preliminary. Nevertheless, as shown in Fig. 3, the two SD patients demonstrated higher expression of TGF-β1, PDGF-AA, and PDGF-BB compared to PD and inevaluable patients.

**Table 5:** Correlative biomarker panel of circulating inflammatory and angiogenic proteins at baseline

Biomarker	*N*	Unit	Median	Range
Ang2	11	pg/ml	345.3	73.1–929.9
BMP-9	11	pg/ml	62.7	30.6–139.9
CD73	11	ng/ml	2.1	0.6–22.8
HER3	11	ng/ml	15.7	12.3–20.6
HGF	11	pg/ml	419.6	83.7–1036.9
ICAM-1	11	ng/ml	454.8	145.0–1042.0
IL-6	11	pg/ml	41.2	15.1–113.6
OPN	11	μg/ml	1.1	0.6–3.0
PDGF-AA	11	pg/ml	106.5	18.1–763.4
PDGF-BB	11	pg/ml	312.8	20.0–1849.4
PlGF	10	pg/ml	18.6	13.1–47.2
SDF1	11	ng/ml	2.4	0.2–5.6
TGF-β1	11	ng/ml	27.7	13.2–160.2
TGF-β2	11	pg/ml	30.4	19.6–104.9
TGFβ-R3	11	ng/ml	86.7	48.6–103.4
TIM-3	10	ng/ml	2.6	1.4–5.1
TIMP-1	11	ng/ml	128.6	42.0–238.5
TSP-2	11	ng/ml	96.9	62.5–315.5
VCAM-1	11	μg/ml	1.4	1.0–5.2
VEGF-A	11	pg/ml	82.6	19.7–400.0
VEGF-D	11	pg/ml	999.8	662.0–3321.0
VEGF-R1	11	pg/ml	281.1	53.2–2063.0
VEGF-R2	11	ng/ml	3.5	1.5–13.0
VEGF-R3	11	ng/ml	301.4	152.0–377.8

## Discussion

This is the first study to investigate the targeted inhibition of ALK1 using PF-03446962 in combination with an anti-angiogenic tyrosine kinase inhibitor. The combination of PF-03446962 and regorafenib demonstrated limited clinical efficacy for patients with metastatic refractory colorectal cancer. The median progression-free survival and median overall survival for patients in our study was lower than previously reported with regorafenib in a large phase 3 clinical trial [12]. While it is possible, the lower starting dose of regorafenib at 120 mg/day may have contributed to the reduced efficacy, the majority of patients treated in the original CORRECT trial required dose modification arguing against the dose affecting outcomes in our study. Furthermore, data from the REDOS trial suggest that lower dose levels are associated with less toxicity and equivalent efficacy [13]. Prior exposure to anti-angiogenic therapies was relatively uniform across the patients treated, with only a single patient having received prior regorafenib. All of the patients had received prior bevacizumab treatment and two patients received ziv-aflibercept as well. The two patients with prolonged disease stabilization raise the possibility of a subset of patients who may benefit from combination VEGF and TGF-β inhibition, or more favorable natural history.

Multiple clinical trials have studied the combination of targeted inhibition of angiogenesis and blockade of the TGF-β axis though with modest results. For example, carotuximab (TRC105), an anti-endoglin monoclonal antibody, has shown promising activity in combination with sorafenib in a phase 1b/2 clinical trial of patients with treatment naïve hepatocellular carcinoma. The combination of the carotuximab and sorafenib resulted in a partial response rate of 25% and reduction in alpha-fetoprotein level in 38% [14]. However, a randomized phase II study of carotuximab in combination with bevacizumab failed to improve PFS in refractory renal cell carcinoma [15]. Carotuximab is currently being evaluated in a phase 3 study with pazopanib in patients with angiosarcoma. Notably, axitinib in combination with delantercept, an ALK1 fusion protein which acts as a trap receptor against BMP 9 and 10, was unable to improve PFS in patients with refractory renal cell carcinoma compared to axitinib plus placebo.

Preliminary biomarker analysis was performed in this study to generate pharmacodynamic data in patients treated with both an ALK-1 inhibitor and a VEGFR inhibitor. We measured 24 biomarkers at baseline and on-treatment in patients treated with combination PF-03446962 and regorafenib to understand the effects of this treatment regimen on key VEGF- and TGF-β-related growth factors. Most observations are consistent with those reported in other studies conducted in the mCRC patient population [16]. However, we did note that certain markers, including OPN, PDGF-AA, and PDGF-BB, were all much higher at baseline compared to patients who were treated in the first-line setting. Whether these differences reflect the underlying biology of this heavily pre-treated population or simply reflects differences in multiplex ELISA assays/components remains unclear.

In other studies involving colorectal cancer patients treated with bevacizumab, PlGF reliably increases, while TSP-2 decreases after initial bevacizumab treatment [10, 16]. Up-regulation of PlGF is a well-defined pharmacodynamic response to VEGF-pathway inhibitors [17]. PlGF belongs to the same family as VEGF members and PlGF up-regulation suggests activation of complementary pathways in response to VEGF signaling blockage [18, 19]. Alternatively, TSP-2 is a natural anti-angiogenic molecule and the initial decreases observed in response to this combination therapy may reflect an attempt to restore balance between pro- and anti-angiogenic factors, as hypothesized in other reports [20]. Interestingly, in this study, we observed that after longer exposure to the combination regimen, PlGF was observed to significantly decrease, while TSP-2 was observed to significantly increase, reversing the change of direction observed at early times (cycle 1) in response to the combination. This may reflect a transition from drug-sensitive to drug-resistant state, as previously reported [20].

Interestingly, we found that TGF-β1, as well as two TGF-β1 downstream effectors, PDGF-AA and PDGF-BB, were noticeably higher in the two SD patients. This preliminary data may suggest that patients with intrinsic high dependence on TGF-β signaling might derive more benefit from drugs targeting TGF-β/ALK1 pathway, or that this is associated with favorable prognosis. Given the limited sample size, these observations need to be confirmed in larger, randomized studies.

## Conclusion

The maximum tolerated dose and recommended phase 2 dosing of combination PF-03446962 and regorafenib was not defined in this study and minimal signal of additive efficacy was observed. Given the limited study size and early closure, conclusions regarding side effect profile are difficult to attain. The correlative angiogenic biomarker analysis performed confirmed expected modulation of the VEGF axis and provides hypothesis-generating data involving potential biomarkers for ALK-1 inhibition. Further investigation is required to fully address whether there is any additive benefit of TGF-β inhibition in combination with anti-angiogenic agents.
